# Geographical disparities in human papillomavirus herd protection

**DOI:** 10.1002/cam4.3125

**Published:** 2020-06-01

**Authors:** Abbey B. Berenson, Jacqueline M. Hirth, Mihyun Chang

**Affiliations:** ^1^ Center for Interdisciplinary Research in Women’s Health Department of Obstetrics & Gynecology University of Texas Medical Branch Galveston TX USA; ^2^ Center for Interdisciplinary Research in Women’s Health University of Texas Medical Branch Galveston TX USA

**Keywords:** geographical disparities, herd immunity, HPV vaccination, human papillomavirus (HPV)

## Abstract

**Background:**

Human papillomavirus (HPV) vaccination has occurred unequally across the United States, potentially contributing to uneven vaccine‐type HPV prevalence between regions. We examined whether emerging vaccine‐related herd protection exhibits regional differences among unvaccinated girls and women.

**Methods:**

We evaluated the prevalence of vaginal HPV among women 14‐59 years of age from 2003 to 2014 using repeated cross‐sectional data from the National Health and Nutrition Examination Survey (NHANES). Women who provided an adequate vaginal swab sample were included. Vaginal prevalence of vaccine‐type HPV (types 6, 11, 16, 18) were examined in four regions of the United States between 2003 and 2014. We examined vaccine‐type HPV prevalence in 2007‐2014 in each US census region among younger participants (14‐34 years old) stratified by vaccination status to determine whether one or both groups contributed to uneven HPV prevalence.

**Results:**

A total of 12 175 participants 14‐59 years of age met inclusion criteria. Vaccine‐type HPV prevalence decreased in all regions. Vaccine‐type HPV varied by region only among unvaccinated 14‐34 year olds, with a higher prevalence in the Midwest (13.8%, 95% confidence interval (CI): 10.7‐17.0) and South (12.5%, 95% CI: 10.2‐14.8) compared to the Northeast (8.9%, 95% CI: 6.5‐11.2). No regional variation in vaccine‐type HPV prevalence was observed among vaccinated participants.

**Conclusions:**

Higher prevalence of vaccine‐type HPV among unvaccinated women in the South and Midwest may contribute to regional disparities in HPV‐related cancer incidence, as emerging herd immunity may not be as strong in those regions.

## Graphical abstract

Uneven HPV vaccination across the US may be contributing to continued geographic disparities in HPV‐related cancers among unvaccinated young women, as emerging herd immunity may not be as strong in regions with lower vaccination rates.

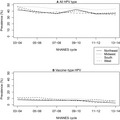

## 1 INTRODUCTION

Human papillomavirus (HPV) is a prevalent infection that causes genital warts and several cancers, including cervical and oropharyngeal cancer. The HPV vaccine series is expected to greatly reduce the morbidity and mortality caused by the virus. In fact, some nations with high HPV vaccine uptake are already reporting reductions in genital warts and abnormal cervical cancer screens.^1^, ^2^ Despite these benefits, the rate of adoption of the vaccine in the United States has been inadequate.^3^ The Advisory Committee for Immunization Practices recommends routine vaccination of adolescents at 11‐12 years of age,^4^ but as of 2018, only 68% of 13‐17 year olds had initiated the HPV vaccine series, and only 51% had completed it.^5^

National surveillance studies indicate that HPV vaccine initiation and completion rates among females are lower in the South and Midwest compared to other regions of the United States.^3^, ^6^, ^7^ This regional inequality among girls and women is a major concern because cervical cancer rates are higher in the South and in some Midwestern states.^8^ Continued low vaccination rates in these regions could further contribute to geographic disparities in HPV infection rates and HPV‐related cancers.

Recently, regional variation in HPV prevalence following the launch of the HPV vaccine in the United States in 2006 was described, with a higher prevalence of vaccine‐type HPV occurring in the South and Midwest than in the West and Northeast in post‐licensure years according to data from the National Health and Nutrition Examination Survey (NHANES).^9^ Additionally, herd immunity is emerging in the US population. These studies demonstrated reduced HPV, genital wart, and precancerous cervical lesion prevalence among unvaccinated groups eligible to receive the vaccine in the United States.^10^, ^11^, ^12^, ^13^, ^14^ Age groups not eligible, or that may have already been exposed before catch‐up vaccination could occur, did not exhibit similar decreases in HPV‐related disease which indicates that HPV vaccination is likely responsible for the emerging herd immunity.^10^ Given these findings and prior reports of regional disparities in HPV vaccination, it is likely that emergent herd protection is also geographically heterogeneous. Therefore, we sought to examine variation in HPV prevalence among vaccinated and unvaccinated women in four regions of the United States during post‐licensure years (2007‐2014) and explore the distribution of herd protection among unvaccinated girls and women in each region using NHANES data.

## 2 MATERIALS AND METHODS

For this study, we utilized the NHANES dataset to conduct a secondary data analysis. NHANES consists of a series of nationally representative, cross‐sectional surveys of the noninstitutionalized US population, conducted in two‐year cycles by the US Centers for Disease Control and Prevention.^15^ Detailed methods have been previously described.^16^ Briefly, NHANES participants complete a household survey and undergo physical exams in a mobile examination center. Females 14‐59 years of age were also asked to self‐collect a vaginal swab for HPV testing. All NHANES swab samples were extracted and tested for types of HPV DNAusing Linear Array HPV genotypying assay (Roche Diagnostics). This data analysis, conducted using NHANES data, was exempted from review by the University of Texas Medical Branch Institutional Review Board. Restricted geographic variables were accessed through the Research Data Center at the National Center for Health Statistics.

The prevalence of HPV by region for six biennial cycles was determined between 2003 and 2014. Inclusion in this study required the following: (a) female, (b) 14‐59 years of age, (c) adequate vaginal swab sample, (d) state of residence, and (e) self‐reported HPV vaccination status for those participating 2007‐2014. HPV results were grouped by type: any of 37 HPV types, vaccine‐types (6, 11, 16, 18), and non‐vaccine types (any HPV types except vaccine‐types). Each HPV type group was transformed into a binary variable, with 0 = no type in the group detected and 1 = at least one type in the group detected.

Participants before the 2007‐2008 cycle were considered unvaccinated, as the vaccine was approved for sale in mid‐2006. Among those who responded to a question asking if they had received at least 1 dose of the HPV vaccine, the proportion of participants who responded “yes” was calculated. Demographics included race/ethnicity, marital status, and health insurance coverage. Behavioral characteristics included cigarette use, sexual behaviors, and alcohol and marijuana use in the past 12 months among 20‐59 year olds. History of gonorrhea and chlamydia were based on self‐report while history of herpes simplex type 2 (HSV2) was based on lab‐confirmed testing and self‐report.

The region of residence for participants in the NHANES dataset was defined by four US census regions, including the Northeast, Midwest, South, and West. Bivariate comparisons were conducted using Rao‐Scott chi‐square tests. For 2007‐2008 through 2013‐2014 cycles, analyses were restricted to participants who responded to whether they had received at least 1 HPV vaccine dose. After examining comparisons among 14‐59 year olds, we grouped the sample into younger (14‐34‐year‐old) and older (35‐59‐year‐old) participants. We included older participants who would not have been eligible for HPV vaccination during the periods examined to determine whether observations were similar or different in both groups. We anticipated that women in the 35 ‐ 59 year old group would not exhibit signs of herd immunity, as most would not have received HPV vaccine doses, and would therefore have different results than the younger age group. Percent change vaccine‐type HPV prevalence between 2003‐2004 and 2013‐2014 cycles was calculated by subtracting 2003‐2004 prevalence from 2013‐2014 prevalence, and then dividing by prevalence of 2003‐2004. Unadjusted logistic regression models were used to compare HPV population adjusted odds ratios (PaORs), which were weighted to produce national estimates as well as adjusted for the complex survey design. Each model was stratified by region. A multivariable logistic regression model was used to calculate adjusted PaORs (aPaORs) for vaccine‐type HPV during post‐licensure cycles, after adjusting for demographics and HPV risk factors. MEC weights were used to account for nonresponse and complex survey methods.^17^ Analyses on the NHANES dataset were conducted using SAS statistical software version 9.4 (SAS Institute, Inc).

## 3 RESULTS

A total of 14 033 females 14‐59 years of age participated across the 6 cycles of NHANES sampling from 2003 to 2014. Of these, 12 182 (86.8%) had valid HPV genotyping results.^7^ After excluding 7 participants without known region of residence, 12 175 participants were eligible for final analyses. For post‐vaccine licensure cycles (2007‐2014), 8025 girls and women 14‐ 59 years old reported their HPV vaccination status.

There were regional differences in several demographic characteristics, including age group, race/ethnicity, and health insurance coverage (Table 1). Regional differences were also observed in behavioral characteristics including cigarette use, sexual activity, number of lifetime partners, vaginal sexual activity, and oral sexual activity. The prevalence of HSV2 also varied by region.

**TABLE 1:** Characteristics of the 14‐ to 59‐year‐old participants by region in the National Health and Nutrition Examination Survey from 2003‐2014 (N = 12,175). (Vaginal sample)

	Frequency (n, w%)										*P*‐value*	
	Overall (N = 12,175)		Northeast^a^ (n = 1947)		Midwest^a^ (n = 2394)		South^a^ (n = 4688)		West^a^ (n = 3146)			
NHANES cycle												
2003‐2006	3929 (32.0)		584 (30.4)		808 (32.2)		1538 (32.3)		999 (32.7)		.96	
2007‐2010	4114 (33.1)		647 (33.7)		912 (36.8)		1544 (32.3)		1011 (30.1)			
2011‐2014	4132 (34.9)		716 (35.9)		674 (31.0)		1606 (35.4)		1136 (37.2)			
Age (y), mean												
14‐34	6201 (42.3)		908 (39.3)		1210 (40.5)		2410 (42.8)		1673 (45.7)		**.02**	
35‐59	5974 (57.7)		1039 (60.7)		1184 (59.5)		2278 (57.2)		1473 (54.3)			
Race												
Hispanic	3413 (15.0)		431 (11.6)		290 (4.9)		1215 (16.3)		1477 (25.4)		**<.0001**	
Non‐hispanic white	4804 (65.1)		877 (71.9)		1433 (80.8)		1501 (56.1)		993 (58.5)			
Non‐hispanic black	2903 (13.2)		431 (10.5)		527 (9.7)		1687 (22.1)		258 (4.9)			
Other	1055 (6.7)		208 (6.0)		144 (4.6)		285 (5.5)		418 (11.2)			
Health insurance coverage												
Private/Public	9103 (80.0)		1628 (87.3)		1940 (85.5)		3284 (74.9)		2251 (77.1)		**<.0001**	
Uninsured	3038 (20.0)		313 (12.7)		450 (14.5)		1390 (25.1)		885 (22.9)			
Missing = 34												
Marital status												
Never married	5872 (61.3)		939 (61.7)		1193 (63.8)		2167 (59.7)		1573 (60.9)		.32	
Married/Living together	1769 (16.1)		283 (15.5)		345 (14.2)		700 (16.8)		441 (17.3)			
Widowed/divorced/separated	3256 (22.6)		534 (22.8)		624 (22.0)		1327 (23.5)		771 (21.8)			
Missing = 1278												
Cigarette use												
Never	7436 (59.7)		1117 (56.0)		1285 (10.2)		2971 (20.4)		2063 (64.1)		**<.0001**	
Former	2138 (21.9)		372 (21.7)		466 (19.8)		749 (15.9)		551 (18.2)			
Current	2422 (18.4)		428 (22.3)		618 (25.9)		893 (21.9)		483 (17.7)			
Missing = 179												
Alcohol use in 12 mo, average number of drinks per week (≥20 yo)												
0	3618 (33.8)		517 (28.0)		600 (27.5)		1558 (40.0)		943 (35.2)		**<.0001**	
≥1	5245 (66.2)		920 (72.0)		1193 (72.5)		1835 (60.0)		1297 (64.8)			
Missing = 899												
Marijuana use^c^												
Never	4854 (47.1)		724 (41.5)		827 (43.7)		1972 (52.8)		1331 (46.0)		**<.0001**	
Former	3122 (42.7)		518 (48.6)		700 (46.3)		1103 (38.4)		801 (41.4)			
Current	951 (10.2)		173 (9.9)		203 (10.0)		320 (8.8)		255 (12.6)			
Missing = 1228												
Ever had sex (any sex)												
No	1235 (7.4)		179 (6.8)		205 (6.8)		451 (7.1)		400 (8.8)		**.05**	
Yes	9793 (92.6)		1553 (93.2)		2003 (93.2)		3793 (92.9)		2444 (91.2)			
Missing = 1147												
Number of lifetime sex partners (any sex) (include only have ever sex)												
0	51 (0.4)		10 (0.6)		5 (0.3)		21 (0.5)		15 (0.4)		**.02**	
1‐2	2959 (27.3)		394 (23.6)		551 (26.5)		1084 (27.2)		930 (30.9)			
≥3	6581 (72.3)		1105 (75.8)		1404 (73.2)		2612 (72.3)		1460 (68.7)			
Missing = 207												
Number of vaginal sex partners in past 12 mo^d^												
0	685 (14.6)		112 (14.8)		120 (13.9)		264 (13.8)		189 (16.2)		.37	
1	3214 (70.1)		543 (72.4)		645 (68.9)		1185 (69.5)		841 (70.7)			
≥2	791 (15.3)		119 (12.8)		164 (17.2)		333 (16.7)		175 (13.1)			
Missing = 230												
HPV vaccine (≥1 dose)^b^												
Yes	1015 (11.2)		208 (13.1)		185 (10.0)		351 (10.7)		271 (11.7)		.28	
No	7010 (88.8)		1114 (86.9)		1379 (90.0)		2719 (89.3)		1798 (88.3)			
Missing = 224												
HPV vaccine dose number, n = 1015 (only for vaccinated women)												
1 dose	187 (15.8)		28 (10.4)		29 (12.8)		77 (18.1)		53 (19.8)		.22	
2 dose	197 (19.9)		43 (19.6)		32 (21.1)		60 (16.3)		62 (24.0)			
3 dose	587 (64.3)		127 (70.0)		117 (66.0)		195 (65.6)		148 (56.2)			
Missing = 44 (excluded from 03‐04 data, and need to consider only for vaccinated women)												
Gonorrhea or chlamydia (self‐reported)												
Positive		448 (2.7)		55 (1.9)		88 (2.8)		214 (3.0)		91 (2.7)		.19
Negative		10 205 (97.3)		1603 (98.1)		2073 (97.2)		3931 (97.0)		2598 (97.3)		
Missing = 1522												
Herpes simplex virus type 2 (lab tested, then self‐reported)												
Positive		1390 (12.4)		217 (11.0)		240 (9.7)		640 (14.9)		293 (12.3)		**.0008**
Negative		9755 (87.6)		1551 (89.0)		1997 (90.3)		3628 (85.1)		2579 (87.7)		
Missing = 1030												

Bolded values indicate significance at *P* < .05.

Abbreviation: w%, weighted percent.

^a^ Northeast states = Connecticut, Maine, Massachusetts, New Hampshire, New Jersey, New York, Pennsylvania, Rhode Island, Vermont; Midwest states = Indiana, Illinois, Iowa, Kansas, Michigan, Minnesota, Missouri, Nebraska, North Dakota, Ohio, South Dakota, Wisconsin; South states = Arkansas, Alabama, Delaware, District of Columbia, Florida, Georgia, Kentucky, Louisiana, Maryland, +Mississippi, North Carolina, Oklahoma, South Carolina, Tennessee, Texas, Virginia, West Virginia; West states = Alaska, Arizona, California, Colorado, Hawaii, Idaho, Montana, Nevada, New Mexico, Oregon, Utah, Washington, Wyoming.

^b^ Data on HPV vaccination were included in survey years 2007‐2014 for females.

^c^ Marijuana data available from 20‐59 yo in 2003‐2006 cycle and 14‐59 yo in 2007‐2014 cycle. Current marijuana use was defined as ≥1 use within the past 30 d.

^d^ Lifetime and recent number of vaginal sex partners included same‐ and opposite‐sex partners.

* Rao‐Scott Chi‐Square test comparing Northeast, Midwest, South, and West.

**FIGURE 1:**
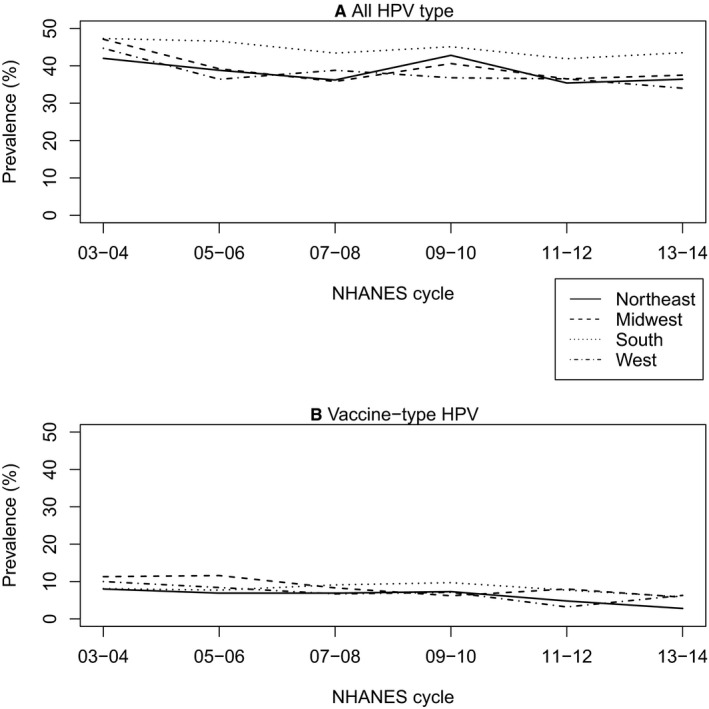
Vaginal human papillomavirus (HPV) prevalence over time, 14‐59 year old women, 2003‐2014. A, All vaccine types include all 37 types that were tested for in the NHANES dataset, including types: 6, 11, 16, 18, 26, 31, 33, 35, 39, 40, 42, 45, 51, 52, 53, 54, 55, 56, 58, 59, 61, 62, 64, 66, 67, 68, 69, 70, 71, 72, 73, 81, 82, 83, 84, CP 6108, and IS39. B, Vaccine type HPV includes types 6, 11, 16, and 18

Prevalence of any HPV type remained stable across time and had similar rates among 14‐59‐year‐old participants in the Northeast, Midwest, and South after the 2005‐2006 cycle (Figure 1A). Between the 2003‐2004 and the 2013‐2014 cycles, vaccine‐type HPV prevalence decreased by 65% in the Northeast, 48.7% in the Midwest, 27.2% in the South, and 37% in the West (Figure 1B).

Among 14‐59‐year‐old participants in the post‐vaccine licensure cycles, we found significant regional differences in the prevalence of non‐vaccine‐type HPV (Table 2). However, after stratifying by vaccination status, there were significant regional differences in HPV prevalence among unvaccinated participants only. Regional differences among the unvaccinated were observed for the prevalence of any type, vaccine type, and non‐vaccine type HPV. No regional differences were observed among vaccinated participants in the post‐licensure cycles.

**TABLE 2:** Human papillomavirus (HPV) prevalence detected through vaginal swab by region and by vaccination status among women aged 14‐59 y, NHANES 2007‐2014 (N = 8025)

	Prevalence (w%, 95% CI)					
	Overall (N = 8025)	Northeast^a^ (n = 1322)	Midwest^a^ (n = 1564)	South^a^ (n = 3070)	West^a^ (n = 2069)	*P* *
Entire sample						
Any HPV types^b^	39.6 (33.6, 45.6)	37.8 (34.1, 41.4)	38.1 (36.9, 39.2)	43.3 (27.6, 59.0)	36.7 (30.6, 42.8)	.06
Vaccine‐type (6, 11, 16, 18)	6.8 (5.8,7.9)	5.4 (2.8, 7.9)	7.1 (4.2, 10.1)	8.1 (5.4, 10.7)	5.8 (0.2, 11.4)	.18
Nonvaccine‐type (any type other than 6, 11, 16, 18)	37.9 (31.3, 44.4)	36.8 (33.0, 40.7)	36.0 (35.0. 37.1)	41.4 (26.8, 56.0)	35.1 (32.9, 37.3)	**.01**
Vaccinated						
Any HPV types^b^	48.7 (44.2, 53.2)	54.5 (45.4, 63.6)	51.1 (43.6, 58.7)	48.5 (39.8, 57.3)	42.1 (34.4, 49.8)	.21
Vaccine‐type (6, 11, 16, 18)	4.9 (3.2, 6.5)	4.5 (0.8, 8.3)	5.9 (1.6, 10.2)	5.8 (2.6, 9.0)	2.9 (0.8, 5.0)	.54
Nonvaccine‐type (any type other than 6, 11, 16, 18)	47.9 (43.4, 52.5)	54.5 (45.4, 63.6)	48.2 (39.5, 57.0)	48.1 (39.4, 56.8)	41.9 (33.9, 49.8)	.27
Unvaccinated						
Any HPV types^b^	38.4 (36.7, 40.2)	35.3 (31.7, 38.8)	36.6 (33.6, 39.7)	42.7 (39.9, 45.5)	36.0 (32.3, 39.7)	**.001**
Vaccine‐type (6, 11, 16, 18)	7.1 (6.3, 7.9)	5.5 (4.3, 6.7)	7.3 (5.9, 8.7)	8.3 (7.0, 9.7)	6.2 (4.5, 7.8)	**.02**
Nonvaccine‐type (any type other than 6, 11, 16, 18)	36.6 (35.0, 38.3)	34.2 (30.6, 37.8)	34.7 (31.6, 37.8)	40.6 (37.9, 43.4)	34.2 (30.8, 37.6)	**.002**
PaOR (95% CI) unvaccinated vs vaccinated						
Any HPV types^b^		**0.46 (0.36, 0.58)**	**0.55 (0.44, 0.70)**	0.79 (0.56,1.12)	0.77 (0.55,1.09)	
Vaccine‐type (6, 11, 16, 18)		1.23 (0.52, 2.89)	1.25 (0.76, 2.05)	1.48 (0.78, 2.81)	**2.20 (1.28, 3.76)**	
Nonvaccine‐type (any type other than 6, 11, 16, 18)		**0.43 (0.34, 0.55)**	**0.57 (0.45, 0.72)**	0.74 (0.52, 1.06)	0.72 (0.51, 1.03)	

Bolded values indicate significance at *P* < .05.

Abbreviations: 95% CI, 95% confidence interval; PaOR, population adjusted odds ratio unadjusted for potential confounders; w%, weighted percent.

^a^ Northeast states = Connecticut, Maine, Massachusetts, New Hampshire, New Jersey, New York, Pennsylvania, Rhode Island, Vermont; Midwest states = Indiana, Illinois, Iowa, Kansas, Michigan, Minnesota, Missouri, Nebraska, North Dakota, Ohio, South Dakota, Wisconsin; South states = Arkansas, Alabama, Delaware, District of Columbia, Florida, Georgia, Kentucky, Louisiana, Maryland, Mississippi, North Carolina, Oklahoma, South Carolina, Tennessee, Texas, Virginia, West Virginia; West states = Alaska, Arizona, California, Colorado, Hawaii, Idaho, Montana, Nevada, New Mexico, Oregon, Utah, Washington, Wyoming.

^b^ HPV types: 6, 11, 16, 18, 26, 31, 33, 35, 39, 40, 42, 45, 51, 52, 53, 54, 55, 56, 58, 59, 61, 62, 64, 66, 67, 68, 69, 70, 71, 72, 73, 81, 82, 83, 84, and IS39.

* Rao‐Scott chi‐Square test comparing Northwest, Midwest, South, and West.

To determine whether emerging herd immunity is apparent, we examined the prevalence of HPV between vaccinated and unvaccinated women by region. Among 14‐34 year olds in post‐licensure cycles, we observed regional differences in the prevalence of vaccine‐type HPV, but not the other groupings of HPV types (Table 3). HPV vaccination among the 14‐34 year olds increased from 10.9% in 2007‐2008 to 34.9% in 2013‐2014, with the greatest increase occurring in the Northeast. However, the vaccination rate within each cycle and in all cycles combined did not vary by US region (Table S2). Conversely, among women 35‐59 years of age, of whom only 1.55% have received the HPV vaccine during 2007‐2014, we observed regional differences for any type, for high‐risk type, for high‐risk non‐vaccine type, and non‐vaccine type HPV in the post‐licensure cycles (Table S1).

**TABLE 3:** Human papillomavirus (HPV) prevalence detected through vaginal swab by region and by vaccination status among women aged 14‐34 y, NHANES 2007‐2014 (N = 3709)

	Prevalence (w%, 95% CI)					
	Overall (N = 3709)	Northeast^a^ (n = 566)	Midwest^a^ (n = 718)	South^a^ (n = 1435)	West^a^ (n = 990)	*P* *
Combined years						
Any HPV types^b^	44.7 (42.4, 47.0)	45.1 (39.6, 50.6)	45.5 (40.3, 50.8)	47.3 (43.9, 50.7)	40.1 (35.6, 44.6)	.10
Vaccine‐type (6, 11, 16, 18)	9.4 (8.4, 10.5)	7.4 (5.1, 9.7)	11.5 (9.1, 13.9)	10.7 (8.7, 12.8)	7.0 (5.0, 9.1)	**.01**
Nonvaccine‐type (any type other than 6, 11, 16, 18)	43.2 (40.8, 45.6)	44.5 (38.7, 50.3)	43.5 (38.3, 48.7)	45.4 (41.8, 49.0)	38.9 (34.4, 43.4)	.16
Vaccinated group						
Any HPV types^b^	49.0 (44.1, 54.0)	55.7 (45.7, 65.6)	49.2 (41.0, 57.5)	48.5 (39.3, 57.7)	43.6 (34.3, 52.9)	.36
Vaccine‐type (6, 11, 16, 18)	4.2 (2.8, 5.6)	4.2 (0.9, 7.4)	4.2 (1.3, 7.0)	4.9 (2.0, 7.9)	3.1 (0.7, 5.4)	.81
Nonvaccine‐type (any type other than 6, 11, 16, 18)	48.6 (43.7, 53.6)	55.7 (45.7, 65.6)	48.3 (40.0, 56.6)	48.2 (39.1, 57.4)	43.3 (33.8, 52.9)	.35
Unvaccinated group						
Any HPV types^b^	43.3 (40.8, 45.8)	40.2 (33.7, 46.7)	44.4 (38.7, 50.0)	46.9 (43.1, 50.7)	39.1 (34.0, 44.2)	.08
Vaccine‐type (6, 11, 16, 18)	11.1 (9.8, 12.4)	8.9 (6.5, 11.2)	13.8 (10.7, 17.0)	12.5 (10.2, 14.8)	8.2 (5.6, 10.8)	**.01**
Nonvaccine‐type (any type other than 6, 11, 16, 18)	41.4 (38.9, 44.0)	39.3 (32.5, 46.2)	42.0 (36.3, 47.7)	44.6 (40.4, 48.7)	37.6 (32.5, 42.8)	.20
PaOR (95% CI) unvaccinated vs vaccinated						
Any HPV types^b^		**0.54 (0.38, 0.75)**	0.82 (0.66, 1.03)	0.94 (0.63, 1.39)	0.83 (0.55, 1.26)	
Vaccine‐type (6, 11, 16, 18)		**2.24 (1.21, 4.14)**	**3.70 (1.21, 4.14)**	**2.74 (1.35, 5.56)**	**2.82 (1.49, 5.34)**	
Nonvaccine‐type (any type other than 6, 11, 16, 18)		**0.52 (0.37, 0.73)**	**0.78 (0.61, 0.98)**	0.86 (0.57, 1.30)	0.79 (0.51, 1.21)	

Bolded values indicate significance at *P* < .05.

Abbreviations: 95% CI, 95% confidence interval; PaOR, population adjusted odds ratio unadjusted for potential confounders; w%, weighted percent.

^a^ Northeast states = Connecticut, Maine, Massachusetts, New Hampshire, New Jersey, New York, Pennsylvania, Rhode Island, Vermont; Midwest states = Indiana, Illinois, Iowa, Kansas, Michigan, Minnesota, Missouri, Nebraska, North Dakota, Ohio, South Dakota, Wisconsin; South states = Arkansas, Alabama, Delaware, District of Columbia, Florida, Georgia, Kentucky, Louisiana, Maryland, Mississippi, North Carolina, Oklahoma, South Carolina, Tennessee, Texas, Virginia, West Virginia; Weststates = Alaska, Arizona, California, Colorado, Hawaii, Idaho, Montana, Nevada, New Mexico, Oregon, Washington, Wyoming.

^b^ HPV types: 6, 11, 16, 18, 26, 31, 33, 35, 39, 40, 42, 45, 51, 52, 53, 54, 55, 56, 58, 59, 61, 62, 64, 66, 67, 68, 69, 70, 71, 72, 73, 81, 82, 83, 84, and IS39.

* Rao‐Scott chi‐Square test comparing Northeast, Midwest, South, and West.

We next sought to determine if emerging herd protection, indicated by declines in vaccine‐type HPV prevalence among the unvaccinated, varies across the United States. We found no regional differences in HPV prevalence for any grouping of HPV types among vaccinated 14‐34‐year‐old participants (Table 3). Among unvaccinated 14‐34 year olds, however, we observed regional variations in vaccine‐type HPV, which was significantly higher among unvaccinated young women compared to vaccinated young women in every region. Among Northeastern participants, those who were unvaccinated had lower prevalence rates of any type HPV compared to those who were vaccinated.

To determine whether these findings were the result of confounding factors, we used multivariable models to calculate aPaORs for demographics and behavioral characteristics among unvaccinated 14‐ to 34‐year‐old women. Vaccine‐type HPV prevalence in unvaccinated participants was lower in the late vaccination periods (2011‐2012 and 2013‐2014) compared to the early period (2007‐2008; Table 4). In a model that included only region and number of lifetime same sex partners, we found that the time effects were magnified compared to the model adjusted with other confounders. (Table S2).

**TABLE 4:** Association of time with vaccine‐type human papillomavirus prevalence after adjusting for demographics and behavioral characteristics among unvaccinated 14‐ to 34‐year‐old women, 2007‐2014 (N = 3,709)

Variable	aPaOR (95% CI)
NHANES cycle	
2007‐2008	Reference
2009‐2010	0.93 (0.66, 1.32)
2011‐2012	**0.59 (0.37, 0.94)**
2013‐2014	**0.53 (0.34, 0.83)**
Race	
Hispanic	Reference
Non‐hispanic white	1.01 (0.64, 1.60)
Non‐hispanic black	1.02 (0.58, 1.79)
Others	1.64 (0.88, 3.04)
Insurance status	
None	Reference
Has insurance	**0.64 (0.44, 0.94)**
Marital status	
Never married	Reference
Married/living with partner	**0.36 (0.24, 0.53)**
Widowed/divorced/separated	0.85 (0.51, 1.41)
Cigarette use	
Never	Reference
Former	1.08 (0.56, 2.08)
Current	**1.74 (1.15, 2.62)**
Alcohol use in 12 mo, average number of drinks per week (≥20 yo)	
0	Reference
≥1	1.21 (0.80, 1.83)
Marijuana use status	
Never	Reference
Former	**1.56 (1.05, 2.33)**
Current	1.28 (0.74, 2.24)
Gonorrhea or chlamydia (self‐reported)	
Negative	Reference
Positive	**3.11 (1.77, 5.49)**
Herpes simplex virus type 2 (tested and self‐reported)	
Negative	Reference
Positive	0.84 (0.52, 1.38)

Note

Bolded values indicate significance at *P* < .05.

Abbreviations: 95% CI, 95% confidence interval; aPaOR, population adjusted odds ratio, adjusted for potential confounding factors as listed in the table.

## 4 DISCUSSION

Human papillomavirus vaccination has been shown to vary across the United States, with the lowest HPV vaccination rates occurring in the South.^7^, ^18^ This study confirms that geographic disparities in HPV vaccination may be problematic, as there were significant geographic differences in vaccine‐type HPV prevalence among unvaccinated women. Regions with lower vaccination rates had the highest prevalence of vaccine‐type HPV among 14‐ to 34‐year‐old unvaccinated women compared to vaccinated women. Similar findings have been reported in a Switzerland‐based modeling study, in which higher HPV type 16 prevalence was associated with regions of lower vaccine uptake.^19^ While this effect may be somewhat mitigated by sexual activity occurring between occupants in different regions of a geographically small nation such as Switzerland, in the larger United States, the majority of residents tend to remain within a single census region. Moves between regions are relatively rare, particularly among populations from lower socioeconomic status groups.^20^ Thus, uneven vaccination rates could contribute to continuing geographic disparities in HPV‐related cancers.^21^, ^22^, ^23^

Our study adds further evidence that herd immunity is emerging among unvaccinated women. A reduction in vaccine‐type HPV prevalence was seen among unvaccinated young women across time in the post‐licensure years, even after controlling for several factors associated with HPV acquisition and persistence. Our findings fit with several prior studies identifying a decrease in HPV prevalence among unvaccinated people in the United States and other countries including Australia and Scotland.^24^, ^25^, ^26^ Although herd immunity in the United States has previously been reported, regional variations were not.^14^ Understanding emerging herd immunity effects at the regional or even local level is important as seemingly strong protection at the national level may belie vulnerability in populations experiencing weaker protection. Recently, clustered outbreaks of measles and pertussis in localized areas with low vaccination rates have caused concerns about how they contribute to perpetuation of these diseases.^27^, ^28^, ^29^, ^30^ It may take more than a decade for the consequences of disparities in HPV immunity to become apparent due to the slow progression from HPV infection to cancer. Nonetheless, the risk of higher regional HPV‐related cancer morbidity and mortality warrants greater attention to regional disparities in HPV prevalence, particularly for regions already presenting with greater incidence of HPV‐related cancers.

The primary strength of this study is that it used repeated cross‐sectional survey data representative of the United States. However, these data also create limitation as they may not be representative of each region. Moreover data on HPV vaccination in NHANES are collected by self‐report, which is subject to recall bias. Furthermore, accuracy of adolescent HPV vaccination reports have been found to vary by race/ethnicity, and could potentially vary by region, which could introduce bias within and between regions.^31^ It is possible that lower vaccine‐type HPV in Northeast and West regions may not be due to herd protection related to higher HPV vaccination rates, but rather due to overall lower circulation of HPV in those regions as a result of differing risk behaviors or other factors.

In summary, this study sheds light on the origin of recently reported regional variation in HPV prevalence in the United States. Only among unvaccinated girls and women did vaccine‐type HPV prevalence vary by region. Notably, odds of vaccine‐type HPV prevalence were higher among unvaccinated young women in the Midwest and South where vaccination rates are not increasing as quickly as in the Northeast. Given the higher prevalence of HPV in the South and Midwest, interventions are needed to increase vaccination rates and to maintain or improve cervical cancer screening in these areas.

## CONFLICTS OF INTEREST

The authors declare no potential conflicts of interest.

## AUTHOR CONTRIBUTIONS

ABB contributed to conceptualization, funding acquisition, project administration, resources, visualization, writing‐original draft, and writing‐review and editing. JMH contributed to conceptualization, formal analysis, methodology, project administration, supervision, writing‐original draft, and writing‐review and editing. MC contributed to conceptualization, formal analysis, methodology, project administration, writing‐original draft, and writing‐review and editing.

## PRECIS

Uneven HPV vaccination across the United States may be contributing to continued geographic disparities in HPV‐related cancers among unvaccinated young women, as emerging herd immunity may not be as strong in regions with lower vaccination rates.

## Supporting information

Table S1

Table S2

## Data Availability

The data that support the findings of this study are available from the Centers for Disease Control and Prevention. Restrictions apply to the availability of these data, which were used under license for this study. Limited data sets are available at https://www.cdc.gov/nchs/nhanes/index.htm. Access to restricted use data is possible with the permission of the National Center for Health Statistics.

## References

[1] Patel C , Brotherton JML , Pillsbury A , et al. The impact of 10 years of human papillomavirus (HPV) vaccination in Australia: what additional disease burden will a nonavalent vaccine prevent? Euro Surveill. 2018;23(41):1700737. 10.2807/1560-7917.ES.2018.23.41.1700737 PMC6194907 30326995

[2] Drolet M , Bénard É , Pérez N , et al. Population‐level impact and herd effects following the introduction of human papillomavirus vaccination programmes: updated systematic review and meta‐analysis. Lancet. 2019;394(10197):497‐509. 31255301 10.1016/S0140-6736(19)30298-3 PMC7316527

[3] Walker TY , Elam‐Evans LD , Yankey D , et al. National, regional, state, and selected local area vaccination coverage among adolescents aged 13–17 years—United States, 2017. MMWR Morb Mortal Wkly Rep. 2018;67(33):909‐917. 30138305 10.15585/mmwr.mm6733a1 PMC6107323

[4] Meites E , Kempe A , Markowitz LE . Use of a 2‐dose schedule for human papillomavirus vaccination—updated recommendations of the advisory committee on immunization practices. MMWR Morb Mortal Wkly Rep. 2016;65:1405‐1408. 27977643 10.15585/mmwr.mm6549a5

[5] Walker TY , Elam‐Evans LD , Yankey D , et al. National, regional, state, and selected local area vaccination coverage among adolescents aged 13–17 years—United States, 2018. MMWR Morb Mortal Wkly Rep. 2019;68(33):718‐723. 31437143 10.15585/mmwr.mm6833a2 PMC6705894

[6] Rahman M , Laz TH , Berenson A . Geographic variation in human papillomavirus vaccination uptake among young adult women in the United States during 2008–2010. Vaccine. 2013;31(47):5495‐5499. 24071591 10.1016/j.vaccine.2013.09.022 PMC3947528

[7] Rahman M , McGrath CJ , Berenson AB . Geographic variation in human papillomavirus vaccination uptake among 13–17 year old adolescent girls in the United States. Vaccine. 2014;32(21):2394‐2398. 24637175 10.1016/j.vaccine.2014.02.097 PMC4062082

[8] Kish JK , Rolin AI , Zou Z , et al. Prioritizing US cervical cancer prevention with results from a geospatial model. Journal of Global Oncology. 2016;2(5):275‐283. 28413829 10.1200/JGO.2015.001677 PMC5389457

[9] Hirth JM , Kuo Y‐F , Starkey JM , et al. Regional variations in human papillomavirus prevalence across time in NHANES (2003–2014). Vaccine. 2019;37(30):4040‐4046. 31182324 10.1016/j.vaccine.2019.06.001 PMC6599727

[10] Gargano JW , Park IU , Griffin MR , et al. Trends in high‐grade cervical lesions and cervical cancer screening in 5 states, 2008–2015. Clin Infect Dis. 2018;68(8):1282‐1291. 10.1093/cid/ciy707 PMC6783904 30137283

[11] Flagg EW , Schwartz R , Weinstock H . Prevalence of anogenital warts among participants in private health plans in the United States, 2003–2010: potential impact of human papillomavirus vaccination. Am J Public Health. 2013;103(8):1428‐1435. 23763409 10.2105/AJPH.2012.301182 PMC4007878

[12] Shing JZ , Hull PC , Zhu Y , et al. Trends in anogenital wart incidence among tennessee medicaid enrollees, 2006–2014: the impact of human papillomavirus vaccination. Papillomavirus Res. 2019;7:141‐149. 30980966 10.1016/j.pvr.2019.04.007 PMC6468146

[13] Flagg EW , Torrone EA . Declines in anogenital warts among age groups most likely to be impacted by human papillomavirus vaccination, United States, 2006–2014. Am J Public Health. 2017;108(1):112‐119. 29161070 10.2105/AJPH.2017.304119 PMC5719685

[14] Berenson AB , Hirth JM , Chang M . Change in human papillomavirus prevalence among U.S. women aged 18–59 years, 2009–2014. Obstet Gynecol. 2017;130(4):693‐701. 28885413 10.1097/AOG.0000000000002193

[15] CfDCaP (CDC) . National Center for Health Statistics (NCHS). National Health and Nutrition Examination Survey Data. U.S. Department of Health and Human Services, Centers for Disease Control and Prevention. https://wwwn.cdc.gov/nchs/nhanes/Default.aspx. Accessed January 2, 2017.

[16] Markowitz LE , Hariri S , Lin C , et al. Reduction in human papillomavirus (HPV) prevalence among young women following HPV vaccine introduction in the United States, National Health and Nutrition Examination Surveys, 2003–2010. J Infect Dis. 2013;208(3):385‐393. 23785124 10.1093/infdis/jit192

[17] National Center for Health Statistics (NCHS) . National Health and Nutrition Examination Survey: questionnaires, datasets, and related documentation. http://www.cdc.gov/nchs/nhanes/. Accessed January 31, 2019.

[18] Hirth JM , Rahman M , Smith JS , Berenson AB . Regional variations in HPV vaccination among 9–17 year old adolescent females from the BRFSS, 2008–2010. Hum Vaccin Immunothe. 2014;10(12):3475‐3483. 10.4161/21645515.2014.980202 PMC4514077 25668660

[19] Riesen M , Garcia V , Low N , Althaus CL . Modeling the consequences of regional heterogeneity in human papillomavirus (HPV) vaccination uptake on transmission in Switzerland. Vaccine. 2017;35(52):7312‐7321. 29126806 10.1016/j.vaccine.2017.10.103

[20] Geronimus AT , Bound J , Ro A . Residential mobility across local areas in the United States and the geographic distribution of the healthy population. Demography. 2014;51(3):777‐809. 24781651 10.1007/s13524-014-0299-4 PMC4129513

[21] Yoo W , Kim S , Huh WK , et al. Recent trends in racial and regional disparities in cervical cancer incidence and mortality in United States. PLoS ONE. 2017;12(2):e0172548. 28234949 10.1371/journal.pone.0172548 PMC5325259

[22] Reiter PL , Fisher JL , Hudson AG , Tucker TC , Plascak JJ , Paskett ED . Assessing the burden of HPV‐related cancers in Appalachia. Hum Vaccin Immunother. 2013;9(1):90‐96. 23143774 10.4161/hv.22389 PMC3667951

[23] Kingsley K , O'Malley S , Ditmyer M , Chino M . Analysis of oral cancer epidemiology in the US reveals state‐specific trends: implications for oral cancer prevention. BMC Public Health. 2008;8(1):87. 18331638 10.1186/1471-2458-8-87 PMC2287178

[24] Brisson M , Bénard É , Drolet M , et al. Population‐level impact, herd immunity, and elimination after human papillomavirus vaccination: a systematic review and meta‐analysis of predictions from transmission‐dynamic models. Lancet Public Health. 2016;1(1):e8‐e17. 29253379 10.1016/S2468-2667(16)30001-9 PMC6727207

[25] Thöne K , Horn J , Mikolajczyk R . Evaluation of vaccination herd immunity effects for anogenital warts in a low coverage setting with human papillomavirus vaccine—an interrupted time series analysis from 2005 to 2010 using health insurance data. BMC Infect Dis. 2017;17(1):564. 28806926 10.1186/s12879-017-2663-7 PMC5557251

[26] Pillsbury AJ , Quinn HE , Evans TD , McIntyre PB , Brotherton JML . Population‐level herd protection of males from a female human papillomavirus vaccination program: evidence from Australian Serosurveillance. Clin Infect Dis. 2017;65(5):827‐832. 29017279 10.1093/cid/cix436

[27] Glasser JW , Feng Z , Omer SB , Smith PJ , Rodewald LE . The effect of heterogeneity in uptake of the measles, mumps, and rubella vaccine on the potential for outbreaks of measles: a modelling study. Lancet Infect Dis. 2016;16(5):599‐605. 26852723 10.1016/S1473-3099(16)00004-9 PMC5859952

[28] Leslie TF , Delamater PL , Yang YT . It could have been much worse: the minnesota measles outbreak of 2017. Vaccine. 2018;36(14):1808‐1810. 29496348 10.1016/j.vaccine.2018.02.086 PMC6626669

[29] Lo NC , Hotez PJ . Public health and economic consequences of vaccine hesitancy for measles in the United States. JAMA Pediatrics. 2017;171(9):887‐892. 28738137 10.1001/jamapediatrics.2017.1695 PMC5710408

[30] Salmon DA , Enger KS , Moulton LH , Halsey NA , Omer SB , Stokley S . Geographic clustering of nonmedical exemptions to school immunization requirements and associations with geographic clustering of pertussis. Am J Epidemiol. 2008;168(12):1389‐1396. 18922998 10.1093/aje/kwn263

[31] Hirth J , Kuo Y‐F , Laz TH , et al. Concordance of adolescent human papillomavirus vaccination parental report with provider report in the National Immunization Survey‐Teen (2008–2013). Vaccine. 2016;34:4415‐4421. 27435385 10.1016/j.vaccine.2016.07.014 PMC4979581

