# Supplementary material for: Geographical disparities in human papillomavirus herd protection
Source: Cancer Med. 2020 Jun 1;9(14):5272–80. doi: 10.1002/cam4.3125 (PMC7367635; doi:10.1002/cam4.3125)
Supplement: Supplementary file 1 — Table S1 [file CAM4-9-5272-s001.docx]

Supplemental table 1. HPV vaccination rate by region among 14–34 year old women across time (N=3,709)

| Year (Cycle) | Total | Vaccination rate (weighted %) | | | | P-value |
| --- | --- | --- | --- | --- | --- | --- |
|  |  | Northeast | Midwest | South | West |  |
| 2007-2008 | 10.9 (6.5, 15.2) | 12.4 (0.0, 31.8) | 10.4 (5.5, 15.2) | 10.3 (3.4, 17.2) | 11.1 (3.8, 18.4) | 0.99 |
| 2009-2010 | 20.2 (17.0, 23.5) | 25.4 (16.5, 34.4) | 22.1 (13.7, 30.5) | 17.6 (12.4, 22.8) | 18.4 (13.9, 22.8) | 0.34 |
| 2011-2012 | 30.1 (25.7, 34.5) | 40.7 (35.1, 46.2) | 31.3 (25.0, 37.7) | 27.7 (21.2, 34.2) | 27.5 (17.8, 37.3) | 0.09 |
| 2013-2014 | 34.9 (29.6, 40.1) | 46.4 (40.8, 52.0) | 34.4 (21.5, 47.4) | 32.8 (22.4, 43.1) | 29.4 (21.9, 36.9) | 0.06 |
|  |  | NHANES cycle 2013-14 compared to cycle 2007-08 | NHANES cycle 2013-14 compared to cycle 2007-08 | NHANES cycle 2013-14 compared to cycle 2007-08 | NHANES cycle 2013-14 compared to cycle 2007-08 |  |
| PaOR (95% CI) |  | **6.10 (1.17, 31.78)** | **4.54 (2.42, 8.39)** | **4.23 (1.89, 9.51)** | **3.34 (1.64, 6.82)** |  |

Bolded values indicate significance at p<0.05.

PaOR = unadjusted population adjusted odds ratio, 95% CI = 95% confidence interval
