# Supplementary material for: Geographical disparities in human papillomavirus herd protection
Source: Cancer Med. 2020 Jun 1;9(14):5272–80. doi: 10.1002/cam4.3125 (PMC7367635; doi:10.1002/cam4.3125)
Supplement: Supplementary file 2 — Table S2 [file CAM4-9-5272-s002.docx]

Supplemental table 2. Comparison of vaccine-type HPV prevalence among 14-34 year olds across time after controlling for region and number of lifetime sexual partners (N=3,709)

| Variable | aPR (95% CI) |
| --- | --- |
| **NHANES cycle** |  |
| 2007-2008 | Reference |
| 2009-2010 | 0.59 (0.33, 1.06) |
| 2011-2012 | **0.28 (0.11, 0.73)** |
| 2013-2014 | **0.24 (0.11, 0.51)** |
| **Region** |  |
| Northeast | Reference |
| Midwest | 1.50 (0.69, 3.23) |
| South | 1.20 (0.67, 2.13) |
| West | 0.83 (0.33, 2.06) |
| **Number of lifetime same sex partners (at least one time )** | |
| 0 | Reference |
| 1-2 | **3.05 (1.67, 5.58)** |
| 3 and over | **2.53 (1.09, 5.89)** |

PR = unadjusted prevalence ratio, 95% CI = 95% confidence interval
